# The Neural Substrates of Memory Suppression: A fMRI Exploration of Directed Forgetting

**DOI:** 10.1371/journal.pone.0029905

**Published:** 2012-01-06

**Authors:** Christine Bastin, Dorothée Feyers, Steve Majerus, Evelyne Balteau, Christian Degueldre, André Luxen, Pierre Maquet, Eric Salmon, Fabienne Collette

**Affiliations:** 1 Cyclotron Research Centre, University of Liège, Liège, Belgium; 2 Department of Psychology: Cognition and Behavior, University of Liège, Liège, Belgium; University of Minnesota, United States of America

## Abstract

The directed forgetting paradigm is frequently used to determine the ability to voluntarily suppress information. However, little is known about brain areas associated with information to forget. The present study used functional magnetic resonance imaging to determine brain activity during the encoding and retrieval phases of an item-method directed forgetting recognition task with neutral verbal material in order to apprehend all processing stages that information to forget and to remember undergoes. We hypothesized that regions supporting few selective processes, namely recollection and familiarity memory processes, working memory, inhibitory and selection processes should be differentially activated during the processing of to-be-remembered and to-be-forgotten items. Successful encoding and retrieval of items to remember engaged the entorhinal cortex, the hippocampus, the anterior medial prefrontal cortex, the left inferior parietal cortex, the posterior cingulate cortex and the precuneus; this set of regions is well known to support deep and associative encoding and retrieval processes in episodic memory. For items to forget, encoding was associated with higher activation in the right middle frontal and posterior parietal cortex, regions known to intervene in attentional control. Items to forget but nevertheless correctly recognized at retrieval yielded activation in the dorsomedial thalamus, associated with familiarity-based memory processes and in the posterior intraparietal sulcus and the anterior cingulate cortex, involved in attentional processes.

## Introduction

Directed forgetting (DF) refers to a deliberate attempt to limit the future expression of specific memory contents [1]–[4]. This active form of forgetting differs from the simple attenuation of memory contents over time and from proactive or retroactive interference. It is involved in many daily memory activities since it allows one to suppress information that is no longer relevant from one's consciousness or to update outdated information. Consequently, intentional forgetting as assessed by DF effects is a desirable and adaptative outcome that can be distinguished from unintentional forgetting and aims at preventing outdated irrelevant information from interfering with current processing and recollection.

Two methods are classically used to study DF effects: the item and list methods. In the item method, participants are presented with words one by one with, after a short delay, the presentation of a “remember” cue (to-be-remembered “TBR” items) or a “forget” cue (to-be-forgotten “TBF” items). Typically, TBR items are better remembered than TBF items, that is, TBR items are better recalled or recognized when participants are subsequently tested on all presented words, regardless of study instructions. In the list method, the participants are generally warned of the status of the items only after a block of items was presented. Some data suggest that each method depends on partially different processes, such as retrieval inhibition for the list method and selective rehearsal or attentional inhibition for the item method [5]–[7]. Currently, the specific mechanisms of directed forgetting and its neural substrates are still discussed. At the cognitive level, two hypotheses have been proposed to explain the DF effect observed with the item method. The first one, the selective rehearsal hypothesis, emphasizes differential encoding and rehearsal of TBR items [6], [8]. According to this hypothesis, when an item is followed by a “remember” cue, participants typically engage in rehearsal and more elaborated encoding than when items are followed by a “forget” cue, naturally making the TBR items more accessible for later remembering. The second hypothesis, the attentional inhibition hypothesis, argues that the item-method DF effect results from the attentional inhibition of TBF items triggered by the “forget” cue [9], [10]. According to this hypothesis, TBF items and/or the rehearsal of these items are assumed to be inhibited just after they are encoded (when the “forget” cue is displayed).

With regard to neuroimaging, only the item-by-item directed forgetting paradigm has been used to explore the neural substrates of (un)successful encoding [11] and (un)intentional forgetting [12]. Reber et al. [11] observed that the anterior ventral portion of the left inferior prefrontal cortex (BA 9), the anterior cingulate (BA 32) and medial superior frontal gyrus (BA 6) exhibited greater activity for TBR than TBF items at encoding. Additionally, the left parahippocampal gyrus and right superior parietal gyrus (BA 7) exhibited greater activity for subsequently remembered words than for subsequently forgotten words. These data show that activity in the ventral prefrontal and superior frontal regions was associated with encoding effort whereas the medial temporal and superior parietal areas were involved in the success of encoding. Wylie et al. [12] observed that intentional forgetting of TBF items was associated with increased activity in the hippocampus and superior frontal gyrus (BA10/11) when contrasted with unintentional forgetting of TBR items, but with higher activity in the medial frontal gyrus (BA10), middle temporal gyrus (BA21), parahippocampal gyrus (BA34/35) and cingulate gyrus (BA31) when contrasted with intentional remembering of TBR items. As a whole, these findings revealed that different brain regions involved in declarative memory are related to intentional forgetting and intentional remembering. These two studies focused on the encoding phase of the item DF paradigm. Recently, Nowicka et al. [13] explored the neural substrates of forgetting effects at encoding and retrieval of neutral and emotionally negative images. They showed that, at encoding, the intention to forget and the success in forgetting negative images were related to more widespread right-hemisphere activations than for neutral images, suggesting greater forgetting effort for emotional materials. At retrieval, forgotten neutral and negative images yielded no cerebral activation. This may indicate that forgetting resulted mainly from inhibitory processes acting at encoding rather than at retrieval.

Finally, the question of inter-individual variability in the ability to overcome the inhibitory/suppression influence of the forget instruction was recently tackled by Nowicka et al. [14] with voxel-based morphometry. In a group of participants with high recognition rates for TBF items, the rate of recognition was related to increased gray matter volume in the left ventrolateral prefrontal cortex (BA 47) and right hippocampus. Such a relationship was not observed for individuals with a low recognition rate for TBF items. Nowicka et al. concluded that these two regions may be part of a neuroanatomical network supporting efficient and successful retrieval of visual information that was not properly encoded and thus difficult to recollect.

The intervention of distinct processes during the DF paradigm is also supported by psycho-physiological data, which demonstrate that the processing of TBF and TBR items is associated with specific event-related potential (ERP) activity. The differential ERP activity was attributed to inhibitory processes of TBF items during encoding [15]–[18] and retrieval [19], [20], and to the involvement of recollection processes for TBR items only [16], [19], [20].

In that context, the main aim of the present study was to explore the neural substrates associated with remembering and forgetting at both the encoding and retrieval stages of a long-term directed forgetting task. Using fMRI, we examined cerebral activation at both encoding and retrieval in relation to memory instructions and behavioural performance in order to apprehend all processing stages that TBR and TBF information undergoes. Whereas Nowicka et al. [13] explored the influence of emotion on the neural bases of directed forgetting, we selected neutral verbal materials so as to focus on basic processes underlying the directed forgetting effect. This will shed further light on the mechanisms of intentional forgetting as well as on the differential richness of the memory trace created for each type of information. Indeed, behavioural studies have highlighted distinct memory processes to operate during the processing of TBR and TBF items. Specifically, TBR information has been shown to lead to elaborated memory traces that can be recollected, while TBF information is shallowly encoded and recognized without recollection of the encoding context [21], [22]. Working memory/executive processes are also considered to intervene in the directed forgetting effect. More particularly, inhibition is the classical explanation of the effect [9] and should operate during the processing of TBF information at encoding. However, another interpretation of the directed forgetting effect consists in selective rehearsal of TBR information [6]. Moreover, intentional forgetting may rely on suppression of irrelevant information, which can be achieved by selection of relevant information before or after its encoding in working memory. Finally, thought suppression should also be observed after presentation of the TBF cue.

Concretely, behavioural recognition data were used to sort encoding and retrieval fMRI event-related responses into 4 conditions based on the combination of memory instruction (to remember vs. to forget) and behavioural outcome (successful vs. unsuccessful recognition). This approach allowed evidencing that processing of TBR and TBF information recruit a very different set of brain regions, compatible with the idea that specific working memory/executive processes induces intentional forgetting and that TBR and TBF information are encoded and retrieved via recollection and familiarity processes respectively.

## Methods

### Ethics Statement

The study was approved by the Ethics Committee of the Faculty of Medicine of the University of Liège, and was performed in accordance with the ethical standards described in the Declaration of Helsinki (1964). All participants gave their written informed consent prior to their inclusion in the study.

### Participants

Seventeen right-handed native French-speaking young adults (8 women), with no diagnosed psychological or neurological disorders, were recruited from the university community. All participants gave their written informed consent prior to their inclusion in the study. Age ranged from 20 to 32 years, with a mean of 24 years.

### Task description

The material consisted of 200 six-letter words selected from the Brulex French database [23]. Two lists of 100 words were created. One list was presented during the study phase; the other was used as foil items during recognition. Items used in the study phase were randomly attributed to two categories of 50 words: (1) items belonging to the category of the words that must be remembered (to-be-remembered or TBR items); (2) items belonging to the category of the words that must be forgotten (to-be-forgotten or TBF items). Allocation of items to the TBR and TBF categories was counterbalanced in order to create two versions of the task, which were administered to participants randomly. Lists of TBR, TBF and foils items were matched for word frequency [*F*(52,2) = 0.001, p>0.5]. Each foil was matched to a target item in order to differ by only one or two letters, corresponding respectively to phonologically similar and dissimilar distractors.

In the study (encoding) phase, the words were individually presented at the centre of a computer screen for 1 second. Each word was followed by either a remember (‘to remember’) or a forget (‘to forget’) cue that remained on the screen for 3 seconds. Participants were asked to read each word mentally and to remember only the words followed by the remember cue (while attempting to forget any word followed by the forget cue). Fifty baseline trials in which the word and instruction were replaced by series of xxxxxx were also presented. The three kinds of trials (TBR, TBF and control) were presented in a pseudo-randomised order, with the restriction that no more than three trials of the same kind were presented in succession. Prior to the beginning of the task, it was stressed that the memory test would only be based on the words labelled as “TBR”. After the learning phase, participants were asked to perform a distraction task that consisted of counting backward in increments of 3 for 60 seconds.

In the retrieval (recognition) phase, the TBR and TBF study words were presented intermingled with an equal number of foils. Each trial of this phase began with the presentation of a word and participants were instructed to press one button if the word had been presented during the study phase (old) – regardless of the previous “remember” or “forget” instruction – and another button if the word had not been presented previously (new). The importance of disregarding the previous “remember” or “forget” instruction was stressed. The baseline condition consisted again in series of crosses associated to random key-press responses. Stimuli remained on the screen until the participant's response, with a maximum allowed time of 5000 msec. If response time was shorter than 3000 msec, a black screen was displayed to ensure that the interval between two successive trials was at least 3000 msec.

### MR acquisition

Functional MRI time series were acquired on a 3T head-only scanner (Magnetom Allegra, Siemens Medical Solutions, Erlangen, Germany) operated with the standard transmit-receive quadrature head coil. Multislice T2*-weighted functional images were acquired with a gradient-echo planar imaging sequence using axial slice orientation and covering the whole brain/most of the brain (32 slices, FoV = 220×220 mm^2^, voxel size 3.4×3.4×3 mm^3^, 30% interslice gap, matrix size 64×64×32, TR = 2130 ms, TE = 40 ms, FA = 90°). The three initial volumes were discarded to avoid T1 saturation effects. 380 scans were obtained in each encoding session while 439 to 565 scans were acquired in the retrieval session. In all sessions, the first three volumes were discarded to account for magnetic saturation effects. For anatomical reference, a high-resolution T1-weighted image was acquired for each subject (T1-weighted 3D magnetization-prepared rapid gradient echo (MPRAGE) sequence, TR = 1960 ms, TE = 4.43 ms, inversion time (TI) = 1100 ms, FoV = 230×173 mm^2^, matrix size = 256×192×176, voxel size = 0.9×0.9×0.9 mm^3^). Head movement was minimized by restraining the participant's head using a vacuum cushion. Stimuli were displayed on a screen positioned at the rear of the scanner, which the participant could comfortably see through a mirror mounted on the standard head coil.

### fMRI analyses

fMRI data were preprocessed and analysed using SPM5 software (Wellcome Department of Imaging Neuroscience, http://www.fil.ion.ucl.ac.uk) implemented in MATLAB (Mathworks, Sherbom, MA). Functional scans were realigned using iterative rigid body transformations that minimize the residual sum of square between the first and subsequent images. They were normalized to the MNI EPI template (voxel size, 2×2×2 mm) and spatially smoothed with a Gaussian kernel with full width at half-maximum (FWHM) of 8 mm (in order to minimize noise and to assure that the residual images conform to a lattice approximation of Gaussian random fields).

For each participant, BOLD responses were modeled at each voxel using a general linear model. For the encoding session, BOLD responses were modeled separately for TBR item recognized as “old” at the retrieval session (TBR-R), TBR items not recognized during retrieval (TBR-F), TBF item subsequently retrieved (TBF-R) and TBF items not retrieved (TBF-F). During the retrieval session, 6 trial types were separately modelled: TBR items correctly recognized (TBR-R) or considered as new items (TBR-F), TBF items categorised as “old” (TBF-R) or “new (TBF-F) items, distractor (i.e., new) items categorised as new (correct rejection, CR) or considered as previously encountered (false alarm, FA).

These ten regressors were modelled as event-related responses. The onsets of the BOLD response for the encoding session were the presentation of the instruction cue (TBR or TBF) and the presentation of the word for the retrieval session. For each trial type, a given item was modeled as a delta function representing its onset. The ensuing vector was convolved with the canonical hemodynamic response function, and used as a regressor in the individual design matrix. Movement parameters estimated during realignment (translations in *x*, *y* and *z* directions and rotations around *x*, *y* and *z* axes) and constant vector were also included in the matrix as a variable of no interest. High pass filter was implemented using a cut off period of 128 s in order to remove the low frequency drifts from the time series. Serial autocorrelations were estimated with a restricted maximum likelihood algorithm using an autoregressive model of order 1 (+white noise). Linear contrasts estimated the simple main effect of each trial type. The resulting set of voxel values constituted a map of *t* statistics SPM[T].

These images were further smoothed (6-mm FWHM gaussian kernel) and entered in two second-level analyses, corresponding to a random effects model, which accounted for inter-subject variance in each contrast of interest. In a first analysis, the four conditions of the encoding session (TBR-R, TBR-F, TBF-R, TBF-F) were entered in an ANOVA with two factors (items status [TBR, TBF] and outcome at retrieval [recognized or forgotten]). A similar ANOVA was performed for the retrieval session (two factors: item status [TBR, TBF] and outcome at retrieval [recognized or forgotten]). Correction for non sphericity due to unequal variance was conducted by covariance component estimation through a expectation–maximization (EM) algorithm [24].

One-sample *t* tests assessed the significance of the effects. More specifically, a simple linear contrast was used to examine the main effect of the forgetting instruction at encoding. For isolating activations related to successful forgetting, encoding and retrieval, the contrast targeting the successful processing of a particular type of event (e.g., TBR) was masked exclusively (p<.05) by the contrast focusing on the successful processing of the other type of event (e.g., TBF) in order to ensure that the activations observed when comparing two conditions were specific to items of interest. The resulting SPM[T] maps were thresholded at *p*<0.001 or *p*<0.005 uncorrected for multiple comparisons with a threshold for minimum spatial extent of 10 contiguous voxels. In order to apprehend the mechanisms of directed forgetting, we explored hypotheses about the cognitive processes and the related cerebral regions that should be involved in each condition by discussing our results in the light of those reported in previous studies and considering brain areas that were very close (no more than 10 mm in the x,y,z directions) to published coordinates of interest. For the cerebral bases of recollection processes, coordinates were taken from a recent meta-analysis [25]. As for more specific processes that we hypothesized to intervene in the task (e.g., effortful deep encoding of verbal material leading to recollection, selection processes…), given that meta-analyses were lacking, we selected studies addressing the neural bases of these processes with materials as close as possible to ours (neutral words, recognition memory tasks). In brief, processing of TBR information was expected to recruit articulatory rehearsal (insula, precentral gyrus, supplementary motor area, Broca's area [26], [27]) and effortul recollection processes (hippocampus, left inferior prefrontal cortex, dorsomedial prefrontal cortex, left inferior parietal cortex, precuneus/posterior cingulate cortex [25], [28]–[34]). With regard to TBF information, their presentation was expected to elicit superficial encoding processes (left dorsolateral prefrontal cortex, right inferior parietal cortex and perirhinal/parahippocampal cortex [29], [35], [36]), inhibitory processes (orbitofrontal cortex, anterior prefrontal cortex; right dorsolateral prefrontal cortex and insula [37], [38]), selection processes (middle frontal gyri, basal ganglia and right parietal cortex [39], [40],[41], [42] and thought suppression (dorsolateral and ventrolateral prefrontal cortex, and anterior cingulate [43]–[45]). Finally, successful retrieval of TBF information should involve regions supporting familiarity-based memory (thalamus, perirhinal cortex, right middle prefrontal cortex, posterior intraparietal sulcus, and anterior cingulate [29], [34], [46], [47]). All stereotactic coordinates refer to the MNI space.

## Results

### Behavioural data

Proportions of old-new recognition responses to TBR, TBF and new items were submitted to an analysis of variance (ANOVA) with item status (TBR, TBF or new) as repeated measure. This analysis revealed a main effect of item status [F(2, 32) = 209.57, p<0.0001], with TBR items being more often recognized than TBF items, and both TBR and TBF items receiving more old responses than new items (TBR items: 83.05±8.49; TBF items: 50.58±14.85; new items: 15.47±8.69; post-hoc HSD Tukey). This pattern corresponds to a significant directed forgetting effect [1]–[4].

The ability to discriminate between each type of items was further determined by means of d' scores [48]. D-prime scores were lower for TBF than TBR items (TBR items: 2.13±0.54; TBF items: 1.12±0.50; t(32) = 5.64, p<.001), suggesting that participants had better memory for TBR than TBF items. Nevertheless, the discrimination of TBF items was significantly above chance (t(32) = 9.20, p<.001). The response criterion measure c, which indicates whether recognition decisions were biased towards conservative or liberal decisions, differed as a function of item status, with participants being more conservative for TBF items than TBR items (TBR items: 0.03±0.28; TBF items: 0.53±0.33; t(32) = −4.80, p<.001).

The use of various strategies was estimated during a subsequent debriefing by means of a 5-point scale (ranging from 1: this strategy was never used by the participant, to 5: this strategy was always used). To memorize TBR items, participants used mainly a rehearsal strategy for one (mean score ± SD: 3.90±1.30) or several (3.71±1.19) items, tried to associate the words to memories or personal events (3.48±1.47). Creation of associations between items to form a short story or a sentence (2.90±1.26) and mental imagery (2.52±1.50) were less often used by the participants. When a forgetting instruction was displayed, participants mainly rehearsed the TBR items presented before (3.33±1.68), tried to think to nothing in particular (3.52±1.47) or to something unrelated to the task (1.86±1.15). As for TBR items, mental imagery unrelated to TBF items (i.e., visualizing an image created with previously presented TBR items) was little used by participants (1.48±1.03).

### fMRI data

#### Encoding phase

1) Cerebral areas associated with directed forgetting.

*Successful forgetting of information to forget (TBF-F>TBF-R).* This analysis did not reveal any significant activation at the selected coordinates.

**Figure 1:**
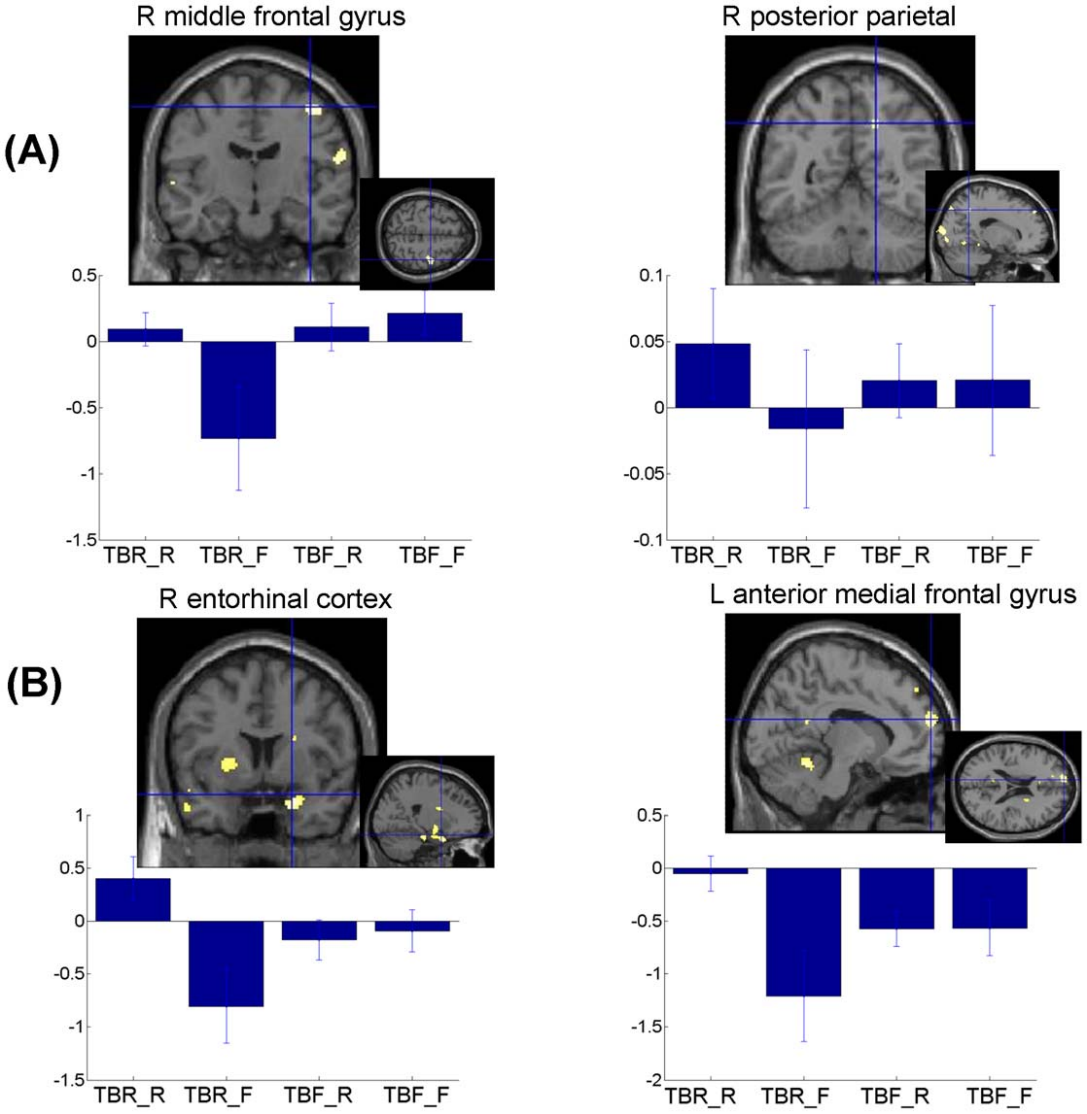
fMRI results for the encoding phase. (**A**). Cerebral areas associated to selection processes. Left: right middle frontal gyrus (larger brain responses for TBF_F than TBR_F information); Right: right posterior parietal (larger brain responses for TBF_F than TBR_F information) (Table 1). (**B**). Cerebral areas associated to encoding of TBR information (Table 2). Left: larger brain response for TBR-R than TBR-F items in right entorhinal cortex. Right: larger brain response for TBR-R than TBF-R information in the anterior medial frontal gyrus. Functional statistical results (p*_uncorrected_*<0.001) are overlaid to a canonical structural image. Activity estimates (arbitrary units) are displayed for the different conditions. *TBR-R*: items associated to a TBR instruction and subsequently recognised; *TBR-F*: items associated to a TBR instruction and subsequently forgotten; *TBF-R*: items associated to a TBF instruction and subsequently recognised; *TBF-F*: items associated to a TBF instruction and subsequently forgotten; New_CR: correct rejection of new items.

*Intentional forgetting of information to forget (TBF-F>TBR-F).* The comparison of TBF items not recognized at retrieval to forgotten TBR items showed increased activity at encoding in the right middle frontal gyrus and the right posterior parietal cortex (Table 1 and Figure 1A).

**Table 1:** Encoding: Cerebral areas associated with directed forgetting.

		MNI coordinates				
Side	Anatomical region	x	y	z	Z	K
*R*	*Middle frontal (BA 4/6) * [*36]*, [40**]	*42*	*−12*	*56*	*3.67*	*90*
R	Postcentral	62	−12	20	3.46	44
R	Superior frontal	18	52	42	3.39	18
R	Inferior orbital frontal	32	24	−24	3.24	15
*R*	*Posterior parietal * [*36*]	*18*	*−54*	*44*	*3.54*	*11*
R	Cuneus	14	−96	8	3.72	447
R	Middle temporal	50	4	−38	3.75	56
L	Superior temporal	−60	−16	2	3.57	19
		−40	−28	6	3.32	19
R	Fusiform gyrus	26	−38	14	3.46	80
R	Lingal gyrus	22	−62	−8	3.64	77
L/R	Superior occipital	−18	−84	34	3.37	19
		20	−84	48	3.43	16
L	Cerebellum (culmen)	−12	−44	−16	3.56	36

Intentional forgetting (TBF-F>TBR-F).

Results at a voxel *P*<0.001, uncorrected for multiple comparisons. L/R = left/right; x, y, z: coordinates (mm) in the stereotactic space defined by the Montreal Neurological Institute (MNI). K = cluster size. Numbers in [] (column 2) correspond to references of studies reporting foci of brain activity close to those observed in the present study (location in x,y,z axes<10 mm).

2) Cerebral areas associated with successful encoding of TBR and TBF items.

*Successful encoding of information to remember: (TBR-R>TBR-F), with exclusive masking by (TBF-R>TBF-F).* This contrast showed regions specifically activated when the information labelled as “to be remembered” was successfully encoded, but not when “to be forgotten” information was successfully encoded. An increase of cerebral activity was observed in the right entorhinal cortex, the anterior medial frontal cortex and the insula bilaterally (Table 2 and Figure 1B).

**Table 2:** Encoding: Cerebral areas associated with successful encoding of TBR items (TBR-R>TBR-F, exclusive masking by TBF-R>TBF-F).

		MNI coordinates				
Side	Anatomical region	x	y	z	Z	K
*L*	*Medial frontal gyrus * [*29*]	*−10*	*62*	*20*	*4.01*	*123*
*L*	*Insula/inferior frontal gyrus * [*26*] ** [*27*]	*−44*	*30*	*−8*	*3.50*	*118*
L	Superior frontal	−16	44	22	3.62	30
L	Superior frontal	−16	34	40	3.82	40
R	Precentral	38	−20	54	3.46	107
L	Precentral	−36	−16	40	3.41	21
L	SMA	−4	−4	72	3.34	15
*R*	*Entorhinal cortex * [*30*]	*22*	*6*	*−26*	*3.82*	*347*
L	Amygdala/hippocampus	−24	−2	−14	3.84	149
R	Superior temporal	64	−6	−4	3.65	75
L	Middle temporal	−56	−6	−20	3.78	141
L	Middle temporal	−58	−32	−16	3.47	67
R	Temporal pole	48	12	−26	3.56	38
L	Putamen	−24	6	4	3.46	57
L	Cerebellum	−8	−50	−14	4.01	115
R	Cerebellum	12	−44	−22	3.93	36
R	cerebellum	34	−72	−48	3.81	16

Results at a voxel *P*<0.001, uncorrected for multiple comparisons. L/R = left/right; x, y, z: coordinates (mm) in MNI space. K = cluster size. Numbers in [] (column 2) correspond to references of studies reporting foci of brain activity close to those observed in the present study (location in x,y,z axes<10 mm).

*Unintentional encoding of information to forget: (TBF-R>TBF-F), with exclusive masking by (TBR-R>TBR-F).* This analysis did not reveal any significant activation at the selected coordinates.

#### Retrieval phase

1) Cerebral areas associated to successful recognition of items following intentional effortful encoding.

**Figure 2:**
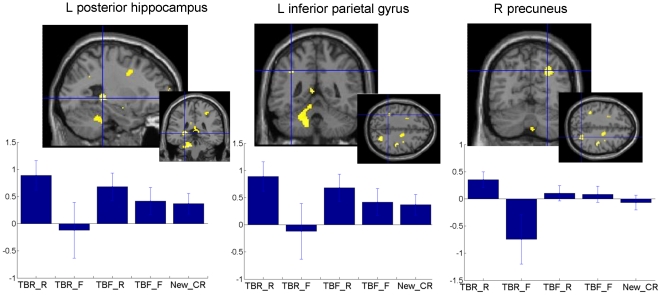
Cerebral areas associated to retrieval of TBR information (**Table 3**). Larger brain responses for TBR-R than TBR-F information in the left posterior hippocampus (left), left inferior parietal cortex (middle) and right precuneus (right). Functional statistical results (p*_uncorrected_*<0.005) are overlaid to a canonical structural image. Activity estimates (arbitrary units) are displayed for the different conditions. *TBR-R*: items associated to a TBR instruction and subsequently recognised; *TBR-F*: items associated to a TBR instruction and subsequently forgotten; *TBF-R*: items associated to a TBF instruction and subsequently recognised; *TBF-F*: items associated to a TBF instruction and subsequently forgotten; *New_CR*: correct rejection of items not presented during the encoding session.

*(TBR-R>TBR-F) with exclusive mask (TBF-R – TBF-F).* This contrast looked at the retrieval success effect specifically for TBR items (i.e., with the exclusion of activation related to successful retrieval of TBF items). At a statistical threshold of p<.001 uncorrected for multiple comparisons at voxel-level, the contrast showed increased cerebral activity in the left posterior hippocampus and the right precuneus. At a more lenient threshold (p<.005 uncorrected), there was also activation in the left inferior parietal regions as well as in the posterior cingulate cortex (see Table 3 and Figure 2).

**Table 3:** Retrieval: Successful retrieval of items following intentional effortful encoding (TBR-R>TBR-F with excusive mask by TBF-R>TBF-F).

		MNI coordinates				
Side	Anatomical region	x	y	z	Z	K
R	Precentral*	34	−8	32	3.40	256
R	Middle frontal*	18	36	−12	3.36	71
L	Middle frontal	−28	6	42	2.80	29
L	Superior frontal	−12	68	18	2.87	102
R	Anterior cingulate*	6	4	28	3.76	606
R	Postcentral*	44	−24	40	3.99	321
R	Superior parietal*	40	−52	66	3.54	92
*R*	*Precuneus * [*28*] *	*26*	*−66*	*38*	*3.75*	*127*
L	Precuneus	−22	−56	30	2.80	26
*L*	*Inferior parietal * [*33*]	*−36*	*−46*	*38*	*3.04*	*35*
*L*	*Posterior cingulate * [*57*]	*−10*	*−36*	*28*	*3.18*	*11*
*L*	*Posterior cingulate * [*34*]	*−6*	*−42*	*10*	*3.09*	*185*
*L*	Posterior hippocampus*	*−26*	*−36*	*0*	*3.80*	*112*
L	Amygdala	−20	−6	−12	3.09	40
L	Uncus	−14	−6	−30	3.08	18
L	Inferior temporal	−58	−50	−20	2.92	21
L	Lingual	−6	−84	−12	3.28	95
R	Pulvinar	24	−26	12	3.07	59
L	Cerebellum*	−14	−38	−32	3.77	569
R	Cerebellum	20	−60	−42	3.29	77

Voxel *P*<0.005 (*p<.001) uncorrected. L/R = left/right. [ ] references of studies with nearby foci of brain activity (location in x,y,z axes<10 mm).

2) Cerebral areas associated to successful recognition of items following unintentional automatic encoding.

**Figure 3:**
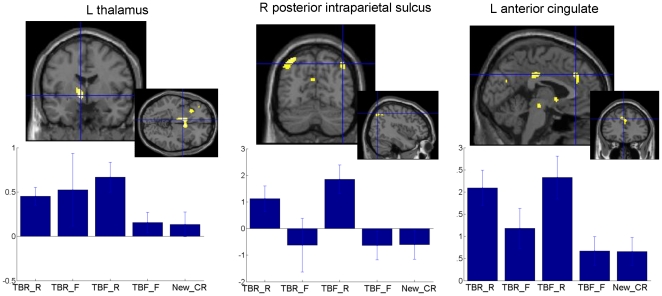
Cerebral areas associated to retrieval of TBF information (**Table 4**). Larger brain responses for TBF-R than TBF-F information in the left thalamus (left), right posterior intraparietal sulcus (middle) and left anterior cingulate (right). Functional statistical results (p*_uncorrected_*<0.001) are overlaid to a canonical structural image. Activity estimates (arbitrary units) are displayed for the different conditions. *TBR-R*: items associated to a TBR instruction and subsequently recognised; *TBR-F*: items associated to a TBR instruction and subsequently forgotten; *TBF-R*: items associated to a TBF instruction and subsequently recognised; *TBF-F*: items associated to a TBF instruction and subsequently forgotten; *New_CR*: correct rejection of items not presented during the encoding session.

*(TBF-R>TBF-F) with exclusive mask (TBR-R-TBR-F).* This contrast looked at the retrieval success effect specifically for items that should have been forgotten, but have nevertheless been recognized (when excluding common activations with successful retrieval of TBR items). It showed increased cerebral activity for TBF-R items in the left dorsomedial thalamus, the right posterior intraparietal sulcus and the anterior cingulate cortex (see Table 4 and Figure 3).

**Table 4:** Retrieval: Successful retrieval of items following unintentional automatic encoding (TBF-R>TBF-F with exclusive mask by TBR-R>TBR-F).

		MNI coordinates				
L	Middle frontal	−44	20	42	3.92	91
L	Insula	−30	18	−20	4.01	56
L	Insula	−30	20	−4	3.68	71
R	Insula	32	20	−10	3.67	51
L	Inferior frontal	−42	42	−2	3.41	32
*R*	*Posterior intraparietal sulcus * [*34*]	*42*	*−70*	*48*	*3.25*	*14*
L	Superior parietal	−36	−66	56	4.06	269
*L*	*Anterior cingulate * [*29*]	*−2*	*34*	*34*	*3.60*	*70*
*L*	*Thalamus * [*47*]	*−8*	*−2*	*−4*	*4.51*	*586*
R	thalamus	6	−8	4	3.37	34
L	Middle cingulate	−2	−22	36	3.35	18

Voxel *P*<0.001 uncorrected. L/R = left/right. [ ] references of studies with nearby foci of brain activity (location in x,y,z axes<10 mm).

## Discussion

The directed forgetting paradigm is frequently used in cognitive psychology and neuropsychology to determine the ability to voluntarily suppress irrelevant information. However, this task is clearly multi-determined and does not only require suppression processes [6], [49]. The current study explored the neural substrates of the processing of TBR and TBF information at both encoding and retrieval in order to shed light on the distinct operations that each type of information undergoes. The analyses of activations related to each type of item as a function of the success of encoding and retrieval showed that different sets of regions are involved in the processing of TBR and TBF information. We will discuss the role of these regions in directed forgetting by reference to the hypothesized underlying cognitive processes.

### Effortful and automatic encoding processes into long-term memory

In this fMRI study, we expected that TBR items would activate brain regions typically associated with effortful encoding processes that promote recollection processes, whereas TBF items would activate brain regions previously found in superficial encoding of information that is later recognised by means of familiarity processes [22].

Consistently, brain activations reflecting encoding success of TBR items, which were not observed for successful encoding of TBF items, included the right entorhinal cortex and the anterior medial prefrontal cortex. The activation of the entorhinal cortex was greater for TBR items subsequently recognised than for any other type of item. Previously, this region was found to be specifically activated by successful associative binding, when participants tried to encode two words by means of a mental image [30]. Thus, it could be involved in the creation of associations between TBR words and semantic or contextual information that participants may generate while elaborating their encoding [29], [46], [50]. The anterior medial prefrontal cortex activated during successful encoding of TBR words corresponds to a region activated during successful deep encoding as compared to successful shallow encoding [29], suggesting that participants may engage additional elaborative (potentially self-related) processing during intentional encoding of TBR items as compared to encoding of TBF items [51].

At retrieval, successful recognition of TBR items, but not successful recognition of TBF items, activated the left hippocampus, left inferior parietal gyrus, the precuneus and the posterior cingulate cortex, regions that are typically related to recollection processes in fMRI studies [25], [28], [33], [34], [46], [52]–[60]. The posterior hippocampus has been related to subjective experience of recollection in a recent meta-analysis [25]. Consistently, previous behavioural findings indicated that recognition of TBR words is more frequently associated with consciously remembering the encoding context than recognition of TBF words [21], [22]. Activations of the precuneus/posterior cingulate are typically observed in episodic memory tasks, but also in tasks involving self-referential processing [61], [62]. Actually, both aspects are interconnected during recollection when one consciously reactivates a personally experienced episode in all its richness (autonoetic consciousness, [63]). With regard to the left inferior parietal cortex, recent models suggest that its role in episodic memory corresponds to the bottom-up capture of attention by information reactivated by the medial temporal lobe. This attentional process may be particularly involved when one retrieves rich contextual details or when one is very confident about ones' memory [64], [65].

No significant activation was found in anticipated coordinates for successful encoding of TBF items. However, the successful recognition of TBF items specifically activated regions sensitive to variable levels of familiarity: the left dorsomedial thalamus and the right posterior intraparietal sulcus. Both regions have been found to be increasingly activated with increasing levels of familiarity [34], [46], [47]. The dorsomedial thalamus is connected to the perirhinal cortex within a system that is thought to mediate familiarity [66]. The intraparietal sulcus has been hypothesized to provide top-down attention to memory, helping to make a memorial decision when the discrimination between old and new items is difficult [64]. Moreover, successful retrieval of TBF items also activated the anterior cingulate cortex. According to Henson et al. (2005), this region may reflect the greater difficulty of retrieving words that underwent superficial encoding as compared to words that were deeply encoded [29]. Altogether, these findings suggest that TBF items were more difficult to discriminate than TBR items and that, when they were successfully recognized, this was mainly because participants felt they were familiar.

### Are inhibitory and/or working memory processes involved in directed forgetting?

Two hypotheses were proposed to explain the DF effect observed with the item method. The first one emphasize selective rehearsal of TBR items [6], [8], [67], [68] while the second one argues that the item-method DF effect results from the attentional inhibition of TBF items triggered by the “forget” cue [9], [10]. Consequently, we will discuss here whether cerebral areas previously associated to inhibitory functioning are involved at encoding during processing of information associated with a TBF cue, or if the presentation of TBR cues involves increased brain activity in areas underlying the articulatory rehearsal process.

No activity was observed in brain areas previously associated to inhibitory abilities [37], [38] or in brain areas associated to the selection of currently relevant memories once information has entered episodic memory [41], [42]. Similarly, the only brain activity in areas previously described as involved in articulatory rehearsal processes [26], [27], [69] was observed in the insula/inferior frontal cortex during successful encoding of information (see Table 2). Consequently, it can be considered that inhibition and selection of relevant episodic memories are not main determinants of the directed forgetting effect as measured by our experimental design, and only a limited role for articulatory rehearsal processes was observed.

We also hypothesized that cerebral areas involved in the selection of information to enter working memory will be more particularly recruited when participants have to suppress TBF information. As expected, activity associated to selection of information to be processed in working memory was observed in right posterior parietal and frontal areas. Observation of parameter estimates showed the lowest activity in these areas for TBR information that was subsequently forgotten. Interestingly, larger activity for TBF-F and TBF-R items was observed in a middle frontal region very close to the area associated to preparatory information filtering processes [39] and this activity is larger for TBF items subsequently forgotten relative to forgotten TBR items, suggesting effectiveness of the filtering process. MacNab and Klingberg [39] proposed that this preparatory activity predicts the extent to which only relevant information is stored, as reflected by parietal storage-related activity. In agreement with this interpretation, cerebral activity in the parietal cortex was characterised by more activity for TBR information that will be latter recognized. However, an alternative explanation for the activation in the posterior parietal lobe during intentional forgetting of TBF information is that this region may prevent information from being encoded in episodic memory following presentation of the TBF instruction. Indeed, it was previously shown that the dorsal parietal cortex mediates voluntary orienting and reorientation of attention [70] and also plays a broader role in the successful formation of episodic memories [71].

Based on the brain regions activated during encoding and retrieval of TBR and TBF items, we tentatively propose that the following complex interplay of cognitive processes operates on TBR and TBF items in order to generate the directed forgetting effect. When a word is followed by a “remember” cue, participants could engage articulatory rehearsal, facilitating the establishment of elaborative encoding. Further, TBR items undergo effortful associative encoding into long term memory that leads, at retrieval, to the reactivation of the rich memory trace created at encoding, a trace which includes the information itself associated with contextual details. In contrast, when a word is labelled “to forget”, cognitive processes related to the selection of information to enter short-term memory come into play because the replacement of information encoding by suppression/selection processes becomes mandatory. Hence, TBF items probably only undergo minimal superficial encoding, so that old TBF items are difficult to discriminate and successful retrieval of TBF happens mainly when the participant merely feels the item was familiar, as suggested by the activation of brain regions involved in familiarity processes and top-down attentional processes during memory retrieval. Thus, the item directed forgetting paradigm may result from the combined action of working memory processes (namely, selection of relevant information to be processed), strategic episodic memory as well as familiarity-based memory processes.

To conclude, this study attempted to delineate, within the same task, brain areas involved in intentional forgetting and (un)intentional remembering. The results obtained here are in agreement with the existence of a network of cerebral areas, reflecting the involvement of several cognitive processes responsible of the DF effect. Further fine-grained studies specifically designed to explore each of these processes and their exact influence on the DF effect should be undertaken. For example, studies using the technique of transcranial magnetic stimulation should allow confirming the role of the areas observed here by examining the kind of errors/responses produced when a site dedicated to a specific process (i.e., the posterior parietal cortex for selection of task-relevant information) is disrupted.
